# The influence of exercise volume and posture on exercise‐induced plasma volume expansion

**DOI:** 10.14814/phy2.15601

**Published:** 2023-02-17

**Authors:** W. Bradley Nelson, James M. Walker, Crystelle Hansen, Kristopher M. Foote, Nathan A. Bexfield, Gary W. Mack

**Affiliations:** ^1^ Department of Exercise Sciences Brigham Young University Provo Utah USA

**Keywords:** albumin, exercise, high‐intensity interval, plasma volume

## Abstract

Acute high‐intensity interval exercise is known to expand plasma volume 24 h after exercise. Upright exercise posture plays a role in expanding plasma volume by influencing lymphatic outflow and redistributing albumin while supine exercise does not. We examined if further upright and weight‐bearing exercises could further promote plasma volume expansion. We also tested the volume of intervals needed to induce plasma volume expansion. To test the first hypothesis, 10 subjects performed intermittent high‐intensity exercise (4 min at 85% V̇_O2max_, 5 min at 40% V̇_O2max_ repeated 8 times) on separate days on the treadmill and cycle ergometer. For the second study, 10 subjects performed four, six, and eight intervals of the same interval protocol on separate days. Changes in plasma volume were calculated from changes in hematocrit and hemoglobin. Transthoracic impedance (*Z*_0_) and plasma albumin were assessed while seated before and postexercise. Plasma volume increased 7.3% ± 4.4% and 6.3% ± 3.5% following treadmill and cycle ergometer exercise, respectively. For four, six, and eight intervals, plasma volume increased by 6.6% ± 4.0%, 4.7% ± 2.6%, and 4.2% ± 5.6%, respectively. The increases in plasma volume were similar for both exercise modes and all three exercise volumes. There were no differences in *Z*_0_ or plasma albumin content between trials. In conclusion, rapid plasma volume expansion following eight bouts of high‐intensity intervals appears to be independent of upright exercise posture (treadmill versus cycle ergometer). Meanwhile, plasma volume expansion was similar after four, six, and eight intervals of cycle ergometry.

## Graphical abstract

Rapid plasma volume expansion after interval exercise: influence of exercise posture and volume.

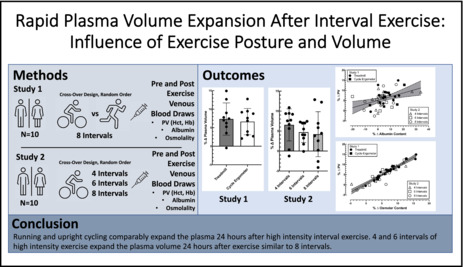

## 1 INTRODUCTION

Plasma volume expansion is a well‐documented adaptation to chronic endurance training (Convertino, 1991). It can also occur more acutely after three consecutive days of high‐intensity interval training and even after just one high‐intensity interval training session of upright cycle ergometry (Gillen et al., 1991; Green et al., 1984). An expanded plasma volume is a beneficial adaptation that provides cardiovascular stability and improved thermoregulatory function in subsequent exercise bouts (Convertino, 1983; Gonzalez‐Alonso et al., 2000). Based on albumin's ability to increase osmotic pressure, shifts in albumin distribution induced by exercise are thought to play a central role in increasing plasma volume after exercise (Gillen et al., 1991). Albumin dynamics are closely tied to posture both at rest and during exercise (Gillen et al., 1991; Nagashima et al., 1999; Wu & Mack, 2001). Specifically, plasma albumin content increases after upright cycle ergometry training (Convertino et al., 1980) and following a single session of high‐intensity intervals (Gillen et al., 1991, 1994; Nagashima et al., 1999). Notably, these changes in plasma albumin are reported to increase within the first hour of recovery from exercise and remain elevated for 22 h after exercise (Nagashima et al., 1999). However, high‐intensity interval exercise performed in the supine posture does not increase plasma albumin content or plasma volume (Nagashima et al., 1999; Ray et al., 1990). Based on the work of Nagashima et al. (1999) and Szabo & Magyar (1967), it is clear that the upright exercise posture provides a better increase in plasma albumin content. Wu & Mack (2001) demonstrated that reducing central venous pressure while in the supine posture by application of lower body negative pressure enhanced the return of albumin to the vascular space when lymphatic flow was elevated following a rapid infusion of saline. The posture‐dependent translocation of albumin presumably elevates plasma albumin content and thereby plasma colloid osmotic pressure, drawing water into the vascular space and increasing plasma volume. The outflow pressure that lymph must overcome to return fluid and albumin to the vasculature is the central venous pressure of the thorax and is easily modified by posture. As such, it is likely that enhanced lymphatic flow during upright exercise contributes to the increase in plasma albumin content.

Ambient temperature, muscle activity, and limb position have been shown to influence the rate of lymph formation, composition, and flow in the human leg (Olszewski, Engeset, Jaeger, et al., 1977). Skeletal muscle contractions, therefore, contribute to the rate of lymphatic flow and subsequent albumin movement (Jacobsson & Kjellmer, 1964; Olszewski & Engeset, 1980; Olszewski, Engeset, & Sokolowski, 1977; Reed, 1985). We conducted two experiments to test the role of exercise posture and volume on plasma volume expansion. First, we hypothesized that upright running, with its corresponding upright posture and greater, involved muscle mass than cycling would elicit higher lymphatic flow rates and enhanced albumin delivery to the plasma than upright cycle ergometry exercise. Previous work has typically used eight intervals of high‐intensity intermittent exercise to elicit an increase in both plasma albumin content and plasma volume (Gillen et al., 1991, 1994; Nagashima et al., 1999, 2001). It is unclear if rapid plasma volume expansion in earlier studies was related to the intensity of the exercise or the volume of exercise performed. We, therefore, performed a second experiment designed to examine the rapid plasma volume expansion following four, six, and eight bouts of high‐intensity cycle ergometer exercise. We tested the hypothesis that eight bouts of high‐intensity exercise were required to produce a significant plasma volume expansion 24 h following exercise.

## 2 METHODS

### 2.1 Subjects and experimental design

Twenty healthy, active college‐age students who were not actively involved in any endurance training program, participated in the current studies. Study 1 compared the plasma volume expansion following eight bouts of high‐intensity interval exercise (4 min at 85% V̇_O2max_ followed by 5 min at 40% V̇_O2max_) on either a cycle ergometer or treadmill and consisted of six males and four female subjects. Study 2 compared the plasma volume expansion following four, six, and eight bouts of high‐intensity interval cycle ergometer exercise V̇_O2max_ (4 min at 85% V̇_O2max_ followed by 5 min at 40% V̇_O2max_) and consisted of five males and five females. Subjects gave written informed consent to the current protocol that was approved by the Brigham Young University Institutional Review Board and conducted in accordance with the Declaration of Helsinki. Subjects for study 1 had a mean ± SD age of 24 ± 3 years, body mass of 71.1 ± 12.3 kg, and height of 172.1 ± 7.9 cm. For study 2, subjects had a mean ± SD age of 23 ± 2 years, body mass of 68.1 ± 15 kg, and height of 172.4 ± 10.7 cm.

For study 1, subjects completed eight bouts of high‐intensity interval exercise on an upright (seated) cycle ergometer (Lode Excalibur), and high‐intensity intervals on a treadmill (Trackmaster, Full Vision Inc) in random order. For study 2, subjects performed three separate high‐intensity interval exercise sessions of four, six, or eight bouts on a Lode cycle ergometer in randomized order. Two female participants took part in both study 1 and study 2. Data from their eight bouts of high‐intensity exercise on a cycle ergometer were used in both study 1 and study 2.

Exercise mode‐specific V̇_O2max_ was determined by indirect calorimetry (Parvo Medics Truemax 2400) using a graded exercise protocol at least 10 days prior to any experimental protocols. Subjects in study 1 had an average relative V̇_O2max_ of 53.2 ± 4.8 mL∙kg^−1^∙min^−1^ (mean ± SD) and an average absolute V̇_O2max_ of 3.82 ± 0.81 L∙min^−1^ on the treadmill. On the cycle ergometer subjects had a relative V̇_O2max_ of 47.5 ± 5.5 mL∙kg^−1^∙min^−1^ and an absolute average V̇_O2max_ of 3.42 ± 0.79 L∙min^−1^. For study 2, subjects had an average relative V̇_O2max_ of 48.6 ± 6.6 mL∙kg^−1^∙min^−1^, and the absolute V̇_O2max_ average was 3.34 ± 0.91 L∙min^−1^ on the cycle ergometer. Treadmill workload (speed and incline) and cycle ergometry workload increments were recorded during maximal exercise tests. Upon completion of the max tests, treadmill speed and incline were noted when the subject was at 85% V̇_O2max_. These values were used during the exercise trials. In study 1, subjects all reached the maximum protocol speed of 3.35 m∙s^−1^ and averaged an incline of 3.4% ± 1.6% when exercising at 85% V̇_O2max_. For cycle ergometry, we calculated the workload at 85% of the maximum wattage achieved on the max test. For study 1, subjects averaged 221 ± 34 W for the intervals, and in study 2, subjects averaged 214 ± 42 W.

Female subjects were studied during the first 5 days after menstruation (days 5–10 of the follicular phase) and all their experimental trials were separated by at least 28 days. Experimental trials for male subjects were separated by at least 10 days.

### 2.2 Experimental protocol

Outside of exercise mode and volume, all protocols and measurements were identical for both study 1 and study 2. Each trial consisted of three consecutive days. Diet and fluid intake were controlled for 16 h on the first experimental day and throughout each trial. On day 2, subjects reported to the lab at 0700 h wearing only shoes and shorts. Female subjects also wore a sports bra. Indoor room temperature averaged 22.6 ± 0.6°C for all subject visits. They were allowed 30 min to consume a fixed breakfast (described below) and 10 mL∙kg body mass^−1^ of water. Upon completion of breakfast, subjects rested in an upright seated posture for 1 h during which time they were instrumented for measurements. A venous catheter was placed in a large antecubital vein while electrocardiogram electrodes and cardiac impedance tape were applied to the surface of the body. Cardiac impedance tape was placed for measurement of *Z*_0_ as an index of thoracic blood volume. The placement of the tape was documented in detail to allow for replicate placement on day 3 and in the subsequent trials. After 60 min, subjects voided their bladders (no sample was collected) and returned to the upright seated posture for another hour to allow equilibration of body fluid compartments. A small blood sample (1 mL) was taken 45 min after being seated and compared with the 60‐minute blood sample to verify a stable baseline for hemoglobin concentration and hematocrit. After 60 min of rest, heart rate, blood pressure, and transthoracic impedance were measured while subjects were seated (1500B EGK Sanborn Series Hewlett Packard Medical Electronics and Minnesota Impedance Cardiograph model 304 B, Surcom Inc). Blood pressure was measured noninvasively with an automated brachial artery arm cuff (Colin 685 STBP Monitor) placed on the arm opposite the venous catheter. Mean arterial pressure was measured using the following equation: mean arterial pressure = (pulse pressure × 0.4) + (diastolic blood pressure) as described here (Bos et al., 2007). Finally, a second‐seated blood sample (20 mL) was drawn. Subjects then voided their bladder again and the entire urine sample was collected to measure volume, osmolality, and electrolytes. Next, the subjects performed a high‐intensity interval exercise protocol (4 min of 85% V̇_O2max_ followed by 5 min of 40% V̇_O2max_). In study 1, the eight high‐intensity intervals were performed on either the treadmill or cycle ergometer in random order on separate visits. In study 2, the four, six, or eight high‐intensity interval exercise bouts were performed in random order on separate visits.

During exercise, heart rate (S810i, Polar Electro) was recorded. Upon completion of the first day of testing, subjects received a 590 mL electrolyte replacement drink, lunch, dinner, and water (10 mL∙kg body mass^−1^) and were then dismissed. They were instructed not to participate in any athletic activity before returning to the lab the next day. On day 3, the procedures were followed exactly as on day 2 except no exercise was performed.

### 2.3 Measurements

#### 2.3.1 Blood analysis

For each blood sample, 0.5 mL of whole blood was used to measure hematocrit and hemoglobin concentration in triplicate. Hematocrit was determined using a microhematocrit technique, and hemoglobin concentration was measured using a cyanmethemoglobin method. Changes in plasma volume were calculated from changes in hematocrit and hemoglobin concentration using the following equation (Dill & Costill, 1974):

%∆PV = {(Hb_pre_/Hb_post_)[(1 − Hct_post_)/(1 − Hct_pre_)]} × 100–100, where ∆PV is the change in plasma volume, pre is the value at baseline, and post is the value at 24 h postexercise, Hb is hemoglobin concentration, and Hct is 0.8736 × hematocrit. We corrected hematocrit values for peripheral sampling by multiplying hematocrit by 0.91 and corrected for trapped plasma by multiplying hematocrit by 0.96 (Convertino et al., 1980, 1981; Gillen et al., 1991; Greenleaf et al., 1979; Hayes et al., 2000). The remaining blood was divided into two vacutainers: lithium heparin and serum for centrifugation. Lithium heparin plasma was used to determine plasma osmolality (freezing point depression, Advanced Osmometer Advanced Instruments), total protein concentration (Pierce BCA), and albumin concentration (BCG Eagle Diagnostic). Serum was used to determine serum electrolyte concentrations using ion‐selective electrodes (Nova Biomedical electrolyte 8+).

#### 2.3.2 Urine analysis

Urine volume was measured with a graduated cylinder. Urine osmolality (freezing point depression) and urine sodium (ion‐selective electrodes) were measured on all urine samples.

### 2.4 Diet intervention

Subjects' diet and fluid intake were controlled for 16 h prior to and throughout the two‐day experimental testing. The diet consisted of five meals: dinner the night before, breakfast, lunch, and dinner on the day of exercise, and breakfast on the day after. Breakfast, lunch, and dinner consisted of 8 kcal∙kg body mass^−1^, 10 kcal∙kg body mass^−1^, and 12 kcal∙kg body mass^−1^, respectively. Subjects were instructed to consume at least 10 mL∙kg body mass^−1^ of water with breakfast and dinner. To aid in rehydration, subjects were given 590 mL of an electrolyte replacement drink upon leaving the laboratory to replace fluid and some electrolytes lost during the high‐intensity interval exercise bout.

### 2.5 Data analysis

We enrolled 10 subjects in each study based on a power analysis that indicated we could detect a true difference in plasma volume of 3% at a *p* < 0.05 statistical significance level. We utilized the Dill/Costill equation (1974) to estimate the change in plasma volume. Direct measurement of absolute plasma volume was not possible because Evans blue dye was unavailable at the time of these studies. Previous work has shown that under the present experimental conditions, the changes in plasma volume are accurately represented by the changes in hematocrit and hemoglobin concentration (Gillen et al., 1991). In order to evaluate changes in plasma albumin and osmolar content we were required to estimate the initial baseline plasma volume for each subject. We chose to use the estimate of 45 mL of plasma per kg of body mass based on the aggregated measurements of numerous studies (Fortney et al., 1983; Gillen et al., 1991, 1994; Haskell et al., 1997, 1998; Mack et al., 1994, 1998; Nagashima et al., 1999, 2001; Nose et al., 1988). Importantly, these studies used the Evans blue dye dilution technique to measure plasma volume across 90 total subjects; collectively, these studies yield a mean plasma volume of 44.5 mL∙kg^−1^. As such, our estimate of plasma volume as 45 mL∙kg^−1^ is a reasonable starting value. Estimates of plasma contents were based on this initial assumption (45 mL∙kg^−1^). The calculated changes in plasma volume were added to this baseline, based on the change in plasma volume percentage calculated from changes in hematocrit and hemoglobin concentrations (Dill & Costill, 1974). Plasma volume and plasma solute contents were reported relative to body mass. In study 1, the baseline blood sample was limited to only determination of hematocrit and hemoglobin concentrations. As such, plasma albumin concentration, plasma albumin content, plasma osmolality, and plasma osmolar content are only present for nine subjects in the data for study 1.

Data are presented as mean ± one standard deviation. For study 1, repeated measures of one‐way ANOVA was used to examine differences between treadmill and cycle ergometer responses. For study 2, repeated measures of one‐way ANOVA was performed to examine differences between the number of high‐intensity intervals performed. Post hoc analysis was performed using the Tukey minimum significant difference test. All analyses were performed using GraphPad Prism software. Statistical significance was established at a confidence level of *p* < 0.05.

## 3 RESULTS

### 3.1 Study 1—Influence of exercise posture on plasma volume expansion

All subjects began each experimental day for each trial fully hydrated as indicated by a mean urine osmolality of 195 ± 15 mOsm∙kg^−1^, there were no significant differences between treadmill exercise and cycle ergometry. Similarly, there were no differences 24 h after exercise in either mode (see Table 1). The mean heart rate during the eight intervals of treadmill exercise was 179 ± 5 beats∙min^−1^ which was similar to the 173 ± 5 beats∙min^−1^ measured during the eight intervals of cycle ergometry. The energy cost of the treadmill and cycle ergometry high‐intensity workouts averaged ≈3418 and 3072 kJ, respectively.

**TABLE 1:** Urine variables.

Variable	Study 1				Study 2					
	Treadmill—8×		Cycle—8×		Cycle—4×		Cycle—6×		Cycle—8×	
	BL	24 h PE	BL	24 h PE	BL	24 h PE	BL	24 h PE	BL	24 h PE
UVol, mL	346 ± 152	297 ± 159	305 ± 159	339 ± 168	273 ± 136	176 ± 89	309 ± 147	277 ± 173	216 ± 81	211 ± 119
[Na]u, mM	46 ± 20	63 ± 25	54 ± 17	57 ± 29	73.1 ± 52.7	87.2 ± 59.7	50 ± 54.0	54.7 ± 37.9	57.8 ± 33.3	62.2 ± 46.5
Na Ex, mmols	14 ± 3.6	17.4 ± 12.2	17.1 ± 10.7	19 ± 12.6	13.9 ± 10.3	12.4 ± 7.4	11.2 ± 6.5	15.1 ± 11.4	11.5 ± 4.6	11.9 ± 8.3
Uosm, mOsm∙kg^−1^	176 ± 72	263 ± 156	167 ± 59	175 ± 54	309 ± 141	369 ± 152	260 ± 151	261 ± 124	303 ± 210	305 ± 141

*Note*: Study 1 Treadmill—8×: eight high‐intensity treadmill intervals, *n* = 10; Cycle—8×: eight high‐intensity cycle intervals, *n* = 10; Study 2 Cycle—4×: four high‐intensity cycle intervals, *n* = 10; Cycle—6×: six high‐intensity cycle intervals; Cycle—8×: eight high‐intensity cycle intervals, *n* = 10; BL, baseline; 24 h PE, 24 h postexercise; UVol, urine volume; [Na]u, urine sodium; Na Ex, urine sodium excretion; Usom, urine osmolality. Study 1: 6 males, 4 females. Study 2: 5 males, 5 females. Means were compared using repeated measures of one‐way ANOVA analysis with Tukey's post hoc test. Values are given as mean ± standard deviation.

**FIGURE 1:**
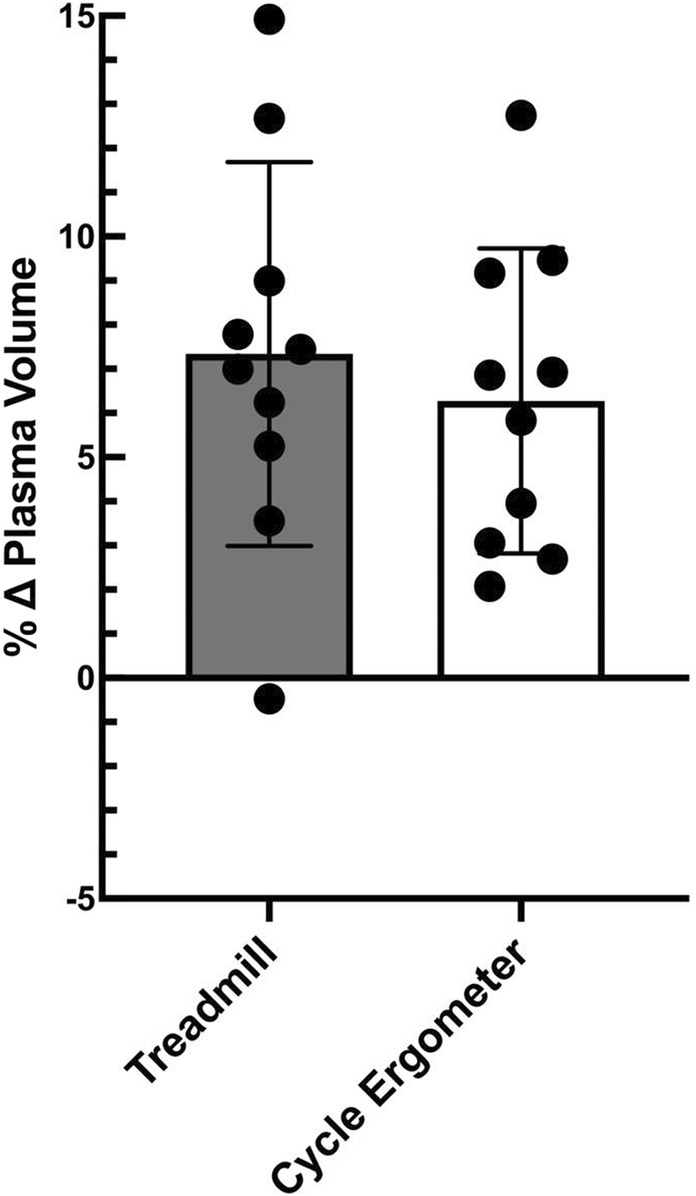
Changes in plasma volume by exercise mode. Values represent means ± standard deviation with individual subject data points shown, means compared using a paired *t* test; *n* = 10, 4 females, 6 males.

Both treadmill exercise and cycle ergometry protocols produced significant increases in plasma volume 24 h postexercise. Plasma volume expansion induced 24 h following treadmill exercise (7.3% ± 4.3%) was similar to the expansion that followed cycle ergometry exercise (6.3% ± 3.5%; Figure 1). The effect of the exercise protocol on plasma variables is shown in Table 2. Both hematocrit and hemoglobin concentration decreased 24 h after treadmill running and cycle ergometry exercise. The decreases in hematocrit and hemoglobin concentration after exercise were similar for both exercise modes.

**TABLE 2:** Plasma variables.

	Study 1				Study 2					
	Treadmill—8×		Cycle—8×		Cycle—4×		Cycle—6×		Cycle—8×	
	BL	24 h PE	BL	24 h PE	BL	24 h PE	BL	24 h PE	BL	24 h PE
Body mass, kg	71.7 ± 13.4	71.6 ± 13.4	71.9 ± 12.8	71.8 ± 12.9	69.2 ± 15.4	70.2 ± 16.1	68.8 ± 15.2	68.8 ± 15.4	68.7 ± 15	68.6 ± 15.8
Hct, %	44.6 ± 3.4	43.3 ± 3.2*	44.8 ± 3.5	43.3 ± 3.5*	43 ± 4.3	43.7 ± 3.5*	43.7 ± 3.5	42.3 ± 3.5*	42.8 ± 4.5	41.8 ± 4.7
Hb, g∙dL^−1^	15.03 ± 1.5	14.26 ± 1.5*	14.84 ± 1.3	14.25 ± 1.1*	14.03 ± 1.67	13.41 ± 1.61*	14.26 ± 1.44	13.9 ± 1.6*	13.99 ± 2.05	13.67 ± 2.2
Δ PV %	—	7.3 ± 4.4*	—	6.3 ± 3.5*	—	6.6 ± 4.0*	—	4.7 ± 2.6*	—	4.2 ± 5.6
[Na]_serum_, mM	139 ± 5.6	138.6 ± 4.1	138 ± 5.1	138.3 ± 4.4	139.3 ± 1.6	139.3 ± 1.2	139.5 ± 1.9	138.9 ± 1.9	138.5 ± 1.7	138.7 ± 2.5
[K]_serum_, mM	3.93 ± 0.33	3.96 ± 0.23	3.9 ± 0.35	3.98 ± 0.22	3.94 ± 0.32	4.03 ± 0.33	3.97 ± 0.29	3.9 ± 0.28	4 ± 0.35	3.89 ± 0.32
Posm, mOsm∙kg^−1^	287 ± 3	287 ± 3	287 ± 4	287 ± 2	287 ± 7	287 ± 5	287 ± 4	286 ± 3	286 ± 3	287 ± 5
TP, g∙dl^−1^	6.75 ± 0.89	6.52 ± 0.5	6.87 ± 0.76	6.90 ± 0.86	7.09 ± 0.83	7.2 ± 1.14	7.34 ± 0.63	7.06 ± 0.58	7.46 ± 0.91	7.31 ± 0.81
Albumin, g∙dl^−1^	5.42 ± 0.39	5.42 ± 0.36	5.33 ± 0.5	5.37 ± 0.62	4.76 ± 0.52	4.77 ± 0.53	4.77 ± 0.64	4.59 ± 0.52	4.91 ± 0.58	4.76 ± 0.58
TP_c_ g∙kg^−1^	3.04 ± 0.39	3.15 ± 0.27	3.09 ± 0.34	3.33 ± 0.44	3.19 ± 0.37	3.51 ± 0.60	3.3 ± 0.28	3.33 ± 0.24	3.36 ± 0.41	3.48 ± 0.47
ALB_c_, g∙kg^−1^	2.44 ± 0.18	2.62 ± 0.16*	2.4 ± 0.23	2.58 ± 0.32	2.14 ± 0.23	2.23 ± 0.28	2.15 ± 0.29	2.16 ± 0.23	2.2 ± 0.26	2.25 ± 0.25
Osm_c_, mOsm	927 ± 171	999 ± 211*	933 ± 177	989 ± 180*	894 ± 200	954 ± 230*	889 ± 194	929 ± 213*	883 ± 193	924 ± 198

*Note*: Study 1: Treadmill—8×: eight high‐intensity treadmill intervals, *n* = 10; Cycle—8×: eight high‐intensity cycle intervals, *n* = 10 (*n* = 9 for Posm, TP, Albumin, TP_c_, ALB_c_, Osm_c_); Study 2: Cycle—4×: four high‐intensity cycle intervals, *n* = 10, (*n* = 9 for TP_C_ and ALB_C_); Cycle—6×: six high‐intensity cycle intervals; Cycle—8×: eight high‐intensity cycle intervals, *n* = 10 (*n* = 9 for TP_C_ and ALB_C_); BL, baseline; 24 h PE, 24 h postexercise; Hct, hematocrit; Hb, hemoglobin; ΔPV, change in plasma volume; [Na]serum, serum sodium; [K]serum, serum potassium; Posm, plasma osmolality; TP, plasma total protein; ALB, plasma albumin. Baseline plasma volume estimated as 45 mL∙kg^−1^. ALB_c_, estimated plasma albumin content; TP_c_, estimated total protein content; Osm_c_, estimated plasma osmolar content. Values are given as mean ± standard deviation. Study 1: 6 males, 4 females. Study 2: 5 males, 5 females. Means were compared using repeated measures of one‐way ANOVA analysis with Tukey's post hoc test.

* *p* < 0.05 different from baseline.

While plasma volume expanded 24 h after exercise for both cycle ergometry and treadmill exercise, subject body mass did not change 24 h after either mode of exercise (see Table 2). We also observed no changes in resting plasma osmolality after exercise for either group. Treadmill exercise produced a significant 7.7% ± 7.2% increase (*p* = 0.0384) in estimated plasma albumin content 24 h following exercise. Following the cycle ergometry exercise, the estimated plasma albumin content increased by 5.9% ± 8.0% but this was nonsignificant (*p* = 0.12). The observed increases in estimated plasma albumin content after exercise were similar for both the treadmill and cycle ergometer protocols. Estimated plasma osmolar content increased after treadmill exercise by 7.6% ± 5.0% (*p* = 0.0134) and cycle ergometry by 6.4% ± 3.3% (*p* = 0.004) with no differences between cycling and treadmill exercises (see Table 2).

Resting transthoracic impedance (*Z*_0_) was similar before and 24 h following each exercise protocol (see Table 3). Urine osmolality, urine sodium concentration and excretion and urine volume before and 24 h following each exercise protocol were also unchanged (Table 1).

**TABLE 3:** Resting cardiovascular variables.

Variable	Study 1				Study 2					
	Treadmill—8×		Cycle—8×		Cycle—4×		Cycle—6×		Cycle—8×	
	BL	24 h PE	BL	24 h PE	BL	24 h PE	BL	24 h PE	BL	24 h PE
SBP, mmHg	114 ± 8	112 ± 11	117 ± 12	113 ± 9	111 ± 8	108 ± 8	112 ± 8	112 ± 8	111 ± 9	114 ± 7
DBP, mmHg	64 ± 10	66 ± 9	70 ± 12	68 ± 8	66 ± 8	63 ± 8	66 ± 6	63 ± 7	64 ± 10	65 ± 7
MAP, mmHg	84 ± 9	85 ± 10	89 ± 11	86 ± 7	84 ± 7	81 ± 7	84 ± 6	83 ± 7	83 ± 9	85 ± 7
HR, beats∙min^−1^	62 ± 12	63 ± 11	60 ± 7	60 ± 6	65 ± 8	64 ± 8	64 ± 11	63 ± 8	66 ± 13	65 ± 10
*Z*_0_, ohms	27.2 ± 4.9	28.1 ± 4.6	27.4 ± 4.2	27.1 ± 4.5	27.6 ± 4.9	27.9 ± 5	28.5 ± 6	28.8 ± 5.1	27.8 ± 4.8	28 ± 4.6

*Note*: Study 1 Treadmill—8×: eight high‐intensity treadmill intervals, *n* = 10; Cycle—8×: eight high‐intensity cycle intervals, *n* = 10; Study 2 Cycle—4×: four high‐intensity cycle intervals, *n* = 10; Cycle—6×: six high‐intensity cycle intervals; Cycle—8×: eight high‐intensity cycle intervals, *n* = 10. 24 h PE, 24 h postexercise; BL, baseline; DBP, diastolic blood pressure; HR, heart rate; MAP, mean arterial blood pressure; SBP, systolic blood pressure; *Z*_0_, transthoracic impedance. Study 1: 6 males, 4 females. Study 2: 5 males, 5 females. Means were compared using repeated measures of one‐way ANOVA analysis with Tukey's post hoc test. Values are given as mean ± standard deviation.

### 3.2 Study 2—Influence of exercise volume on plasma volume expansion

Body mass was similar prior to and 24 h after exercise for all three cycle ergometer trials (see Table 2). The mean heart rate during each exercise protocol averaged 174 ± 9 beats∙min^−1^, 172 ± 8 beats∙min^−1^, and 174 ± 8 beats∙min^−1^ for four, six, and eight bouts of intervals respectively. The total energy cost of the four, six, and eight bouts of high‐intensity cycle ergometer exercise in study 2 averaged 1440, 2160, and 2880 kJ, respectively.

**FIGURE 2:**
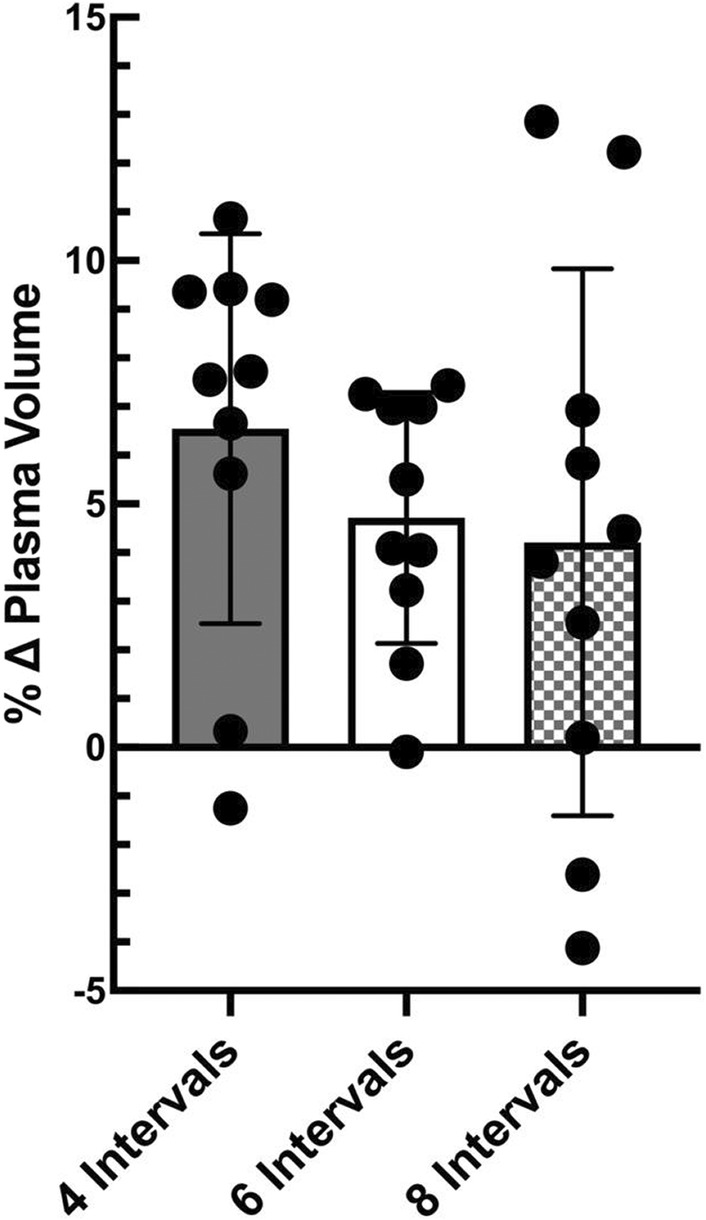
Changes in plasma volume by exercise volume. Values represent means ± standard deviation with individual subject data points shown, means compared using repeated measures of one‐way ANOVA; *n* = 10, 5 females, 5 males.

Plasma volume increased (*p* < 0.01) 24 h after four (6.6% ± 4.0%) and six (4.7% ± 2.6%) bouts of high‐intensity interval exercise. However, eight bouts of high‐intensity intervals did not increase (4.2% ± 5.6%) 24 h later (*p* = 0.26). However, the changes in plasma volume were similar for all three exercise protocols (Figure 2). Hematocrit and hemoglobin decreased 24 h after four and six bouts of interval exercise (Table 2, *p* < 0.05). Eight bouts of interval exercise failed to significantly reduce hemoglobin or hematocrit 24 h after exercise (hematocrit *p* = 0.0806, hemoglobin *p* = 0.517).

*Z*_0_ and plasma albumin content were similar before and after exercise as well as between trials (Tables 2 and 3). Osmolar content significantly increased (*p* < 0.05) 24 h following four (6.6% ± 5.8%) and six bouts (4.5% ± 2.2%) of high_intensity intervals but not after eight bouts (6.0% ± 4.6%, *p* = 0.14). However, there was no difference in osmolar content increases among the three groups. Resting renal function as measured by urine osmolality, urine sodium concentration and excretion and urine volume were similar before and after exercise for all of the exercise protocol trials (Table 1).

**FIGURE 3:**
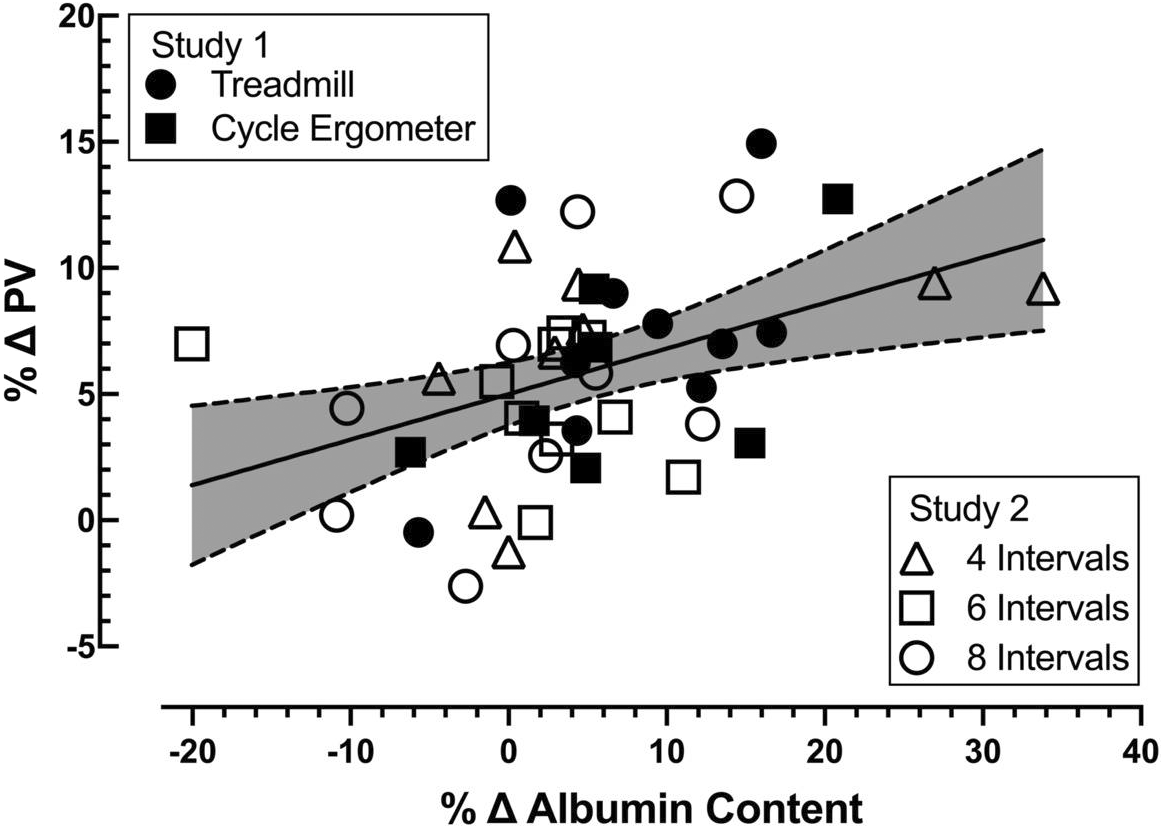
Relationship between the percent change in plasma volume 24 h following exercise and the percent change in estimated plasma albumin contents. Individual data for each subject for each exercise mode and each subject for the three exercise volumes performed. Best‐fit line by least squares linear regression, shading between the dotted lines represents the 95% confidence interval. All groups combined (shown), *r* = 0.4254, *p* = 0.0036; *n* = 45, 16 females, 19 males.

**FIGURE 4:**
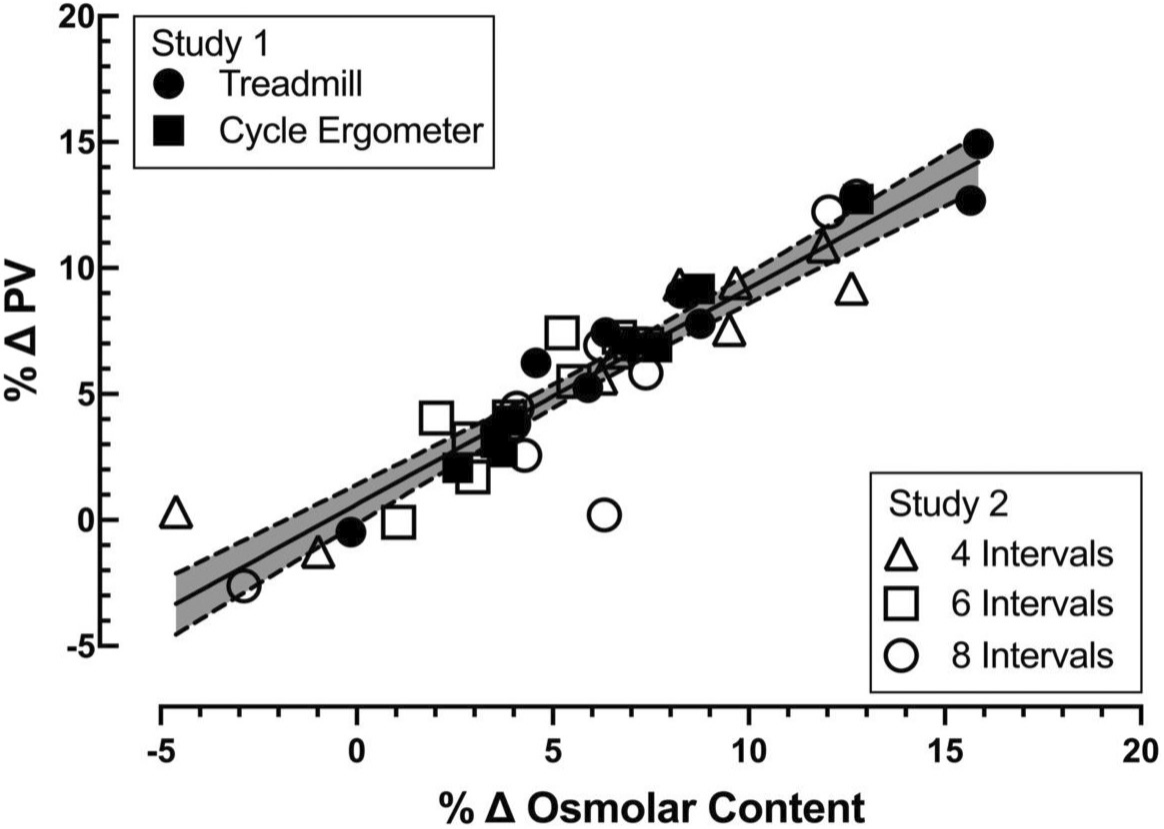
Relationship between the percent change in plasma volume 24 h following exercise and the percent change in estimated plasma osmolar content. Individual data for each subject for each exercise mode and each subject for the three exercise volumes performed. Best‐fit line by least squares linear regression, shading between the dotted lines represents the 95% confidence interval. All groups combined (shown), *r* = 0.9303, *p* < 0.0001; *n* = 45, 16 females, 19 males.

To examine the relationship between the change in plasma albumin and osmolar content on plasma volume expansion we pooled data from all five exercise protocols from study 1 and study 2. The magnitude of plasma volume expansion (∆%) following the exercise was proportional to the change in estimated plasma albumin content (∆%; *p* = 0.0036, Figure 3). The change in plasma volume (∆%) was also positively correlated with the change in estimated plasma osmolar content (∆%) 24 h after the high‐intensity exercise bouts (*p* < 0.0001, Figure 4).

## 4 DISCUSSION

We report several principal findings of the present studies. First, plasma volume expansion 24 h after high‐intensity interval exercise was similar following high‐intensity intermittent treadmill running and cycle ergometry exercise. Second, plasma volume expansion 24 h after expending 1440 kJ (four bouts of high‐intensity cycle ergometer exercise) was similar to expending 2880 kJ (eight bouts of high‐intensity cycle ergometer exercise). Third, the magnitude of plasma volume expansion following high‐intensity intermittent exercise was proportional to the increase in plasma albumin and osmolar content.

Rapid plasma volume expansion within 24 h following high‐intensity interval exercise has been reported to occur due to an increase in plasma albumin content (Gillen et al., 1991; Nagashima et al., 1999). This increase in plasma albumin content acts to draw interstitial water into the vasculature space. Intense exercise elevates plasma albumin via a redistribution of albumin stores from the interstitial space to the vascular compartment by way of the lymphatics with a concomitant reduction in albumin transcapillary escape (Convertino et al., 1980; Haskell et al., 1997). Rapid increases in albumin synthesis do not appear to be critical to acute plasma volume expansion but likely contribute to the increased albumin content associated with chronic exercise training (Nagashima et al., 2000; Yang et al., 1998). During exercise, the ability of the lymphatic circulation to return albumin to the vascular space is influenced by several factors including posture, exercise intensity, and the involved muscle mass (Jacobsson & Kjellmer, 1964; Olszewski & Engeset, 1980; Olszewski, Engeset, & Sokolowski, 1977; Reed et al., 1985). Any exercise that can increase lymphatic return should facilitate the movement of albumin from the peripheral tissues to the cardiovascular system contributing to an increase in plasma volume following intense exercise. We hypothesized that fully upright and weight‐bearing treadmill running would positively influence lymphatic outflow, increase albumin translocation to the vasculature, and elicit a greater plasma volume expansion than upright cycling. However, our data did not support this hypothesis. We noted similar plasma volume expansions with cycle ergometry and treadmill running. The exercise intensity and posture were similar enough that no differences were detected in any of the relevant measures we report.

The original high‐intensity intermittent (eight intervals) cycle ergometry protocol has been used to induce plasma volume expansion 24 h after exercise (Gillen et al., 1991, 1994; Nagashima et al., 1999). We examined this model to see if fewer intervals could induce a similar expansion of plasma volume. We compared four and six intervals to eight intervals and found no differences in the increase in plasma volume after exercise. Performing eight 4‐minute intervals at 85% V̇_O2max_ interspersed with 5 min at 40% V̇_O2max_ represents 32 min of high‐intensity exercise and 72 total min of exercise. It is interesting to note that four intervals achieved the same plasma volume expansion as eight intervals after only 16 min of high‐intensity work and 36 min of total exercise. Freund et al. reported that well‐trained subjects produced a significant plasma volume expansion 1 h following a single treadmill maximal effort (seven stages, 2 min for each stage; Freund et al., 1987). This group also observed, like others, that plasma volume decreased during exercise. However, the untrained subjects were only able to recover their plasma volume back to baseline 1 h after exercise, similar to other studies of untrained subjects (Gillen et al., 1991; Nagashima et al., 1999). Based on our current findings, an exercise protocol that includes at least 16 min of high‐intensity exercise and expends at least 1440 kJ is sufficient to stimulate an increase in plasma volume (≈4%–8%) within 24 h after exercise in untrained subjects.

We used transthoracic impedance (*Z*_0_,) as an indicator of changes in thoracic blood volume (Wu & Mack, 2001). An increase in *Z*_0_ would be associated with a decrease in thoracic blood volume and central venous pressure and thereby promote the lymphatic return of proteins to the vasculature by reducing lymphatic outflow pressure (Brace, 1989; Nagashima et al., 1999; Szabo & Magyar, 1967; Wu & Mack, 2001). In supine subjects, the application of lower body negative pressure reduced *Z*_0_ and lymphatic outflow pressure and subsequently increased lymphatic delivery of albumin to the vascular compartment (Wu & Mack, 2001). We did not observe any change in *Z*_0_ at rest 24 h after exercise for any combination of posture or exercise volume. As such, lymphatic delivery of albumin to the vascular compartment during recovery from high‐intensity exercise in our study could not be attributed to noticeable differences in lymphatic outflow pressure. Since the increase in plasma albumin content was similar across four, six, and eight bouts of high‐intensity exercise, we cannot attribute the increases in albumin content to exercise volume‐altering lymphatic return of albumin to the vasculature.

We observed that the rapid increase in plasma volume following high‐intensity intermittent exercise was associated with an increase in plasma osmolar content. Both increased colloid oncotic pressure (i.e., increased plasma albumin content) and increased osmolar content contribute to plasma volume expansion after high‐intensity intermittent exercise. Both these systems exert osmotic pressure to draw water into the vasculature compartment. In the present study, the increase in plasma volume following high‐intensity intermittent exercise was significantly correlated with both the change in plasma albumin content (Figure 3, *p* = 0.0036) and the increase in plasma osmolar content (Figure 4, *p* < 0.0001). The increase in plasma albumin content supports the idea that a selective expansion of the plasma volume occurs following high‐intensity intermittent exercise. Conversely, the increase in osmolar content supports the idea that the expansion of the extracellular fluid space via osmotic forces is also an important contributor to the expansion of plasma volume via its impact on extracellular fluid volume.

Several studies have attempted to uncover the physiological mechanism(s) driving rapid plasma volume expansion following high‐intensity exercise (Gillen et al., 1991; Haskell et al., 1997; Mack et al., 1998; Nagashima et al., 1999; Wu & Mack, 2001). The first observation was that a selective expansion of plasma volume immediately after exercise was associated with an increase in plasma albumin content (Gillen et al., 1991). They noted that the increase in plasma albumin accounted for 90% of the increase in total body water. Nagashima et al. (1999) clearly demonstrated that rapid plasma volume expansion following intense exercise was due primarily to the increase in plasma albumin content. This response was blunted when exercise was performed in the supine posture. The rapid expansion of plasma volume following high‐intensity exercise is allowed to happen because reflex endocrine and renal responses to an increase in blood volume are attenuated immediately following exercise for approximately 44 h (Gillen et al., 1994). More importantly, the acute renal response to high‐intensity exercise compliments the role of albumin in the expansion of plasma volume (Nagashima et al., 2001). Both Mack et al. (1998) and Haskell et al. (1997) found that changes in microvascular forces that control transcapillary albumin flux change in a direction to support reduced transcapillary albumin flux. Specifically, earlier work has identified a negative association between reductions in transcapillary escape rate and increased plasma volume (Haskell et al., 1997). The present study adds to this body of knowledge by demonstrating that albumin translocated into the vascular compartment following only four bouts of high‐intensity exercise is sufficient to expand plasma volume to the same magnitude as eight bouts of high‐intensity exercise. One interpretation of these data is that the pool of albumin available for rapid translocation into the vascular space must respond early during upright high‐intensity exercise and is dependent on exercise intensity, not volume.

Hypervolemia is a beneficial and reproducible adaptation to endurance exercise. Notably, an augmented blood volume improves thermoregulation and cardiac stability during exercise (Wyndham et al., 1968, 1976). Cardiac output and aerobic power are also improved with an expanded blood volume (Coyle et al., 1986). A larger blood volume is a function of increased plasma volume and an expanded erythrocyte volume (Brotherhood et al., 1975) and nearly all of the blood volume increase seen within the first 14 days of endurance training is attributed solely to the elevated plasma volume (Sawka et al., 2000). The increase in erythrocyte volume lags 2–3 weeks behind the initial plasma expansion (Sawka et al., 2000). This initial rapid expansion of plasma volume will provide immediate support to cardiovascular stability during subsequent exercise bouts. Our current findings contribute to our understanding of the stimuli that are sufficient to induce rapid plasma volume expansion in untrained individuals.

Our work does have some limitations. First, we were unable to measure plasma volume using Evans blue dye dilution and therefore used a reasonable estimate of resting plasma volume for our subject pool. Second, we did not measure lymphatic delivery of albumin to the vascular space directly. Rather we measured the increase in plasma albumin content as a surrogate of lymphatic delivery of albumin to the vascular space. Finally, our experimental design did not allow us to identify the threshold of exercise volume required to expand the plasma after exercise at a fixed exercise intensity. However, we did observe that four bouts of intervals increased plasma after exercise to a similar extent as eight bouts.

In conclusion, plasma volume expansion following high‐intensity interval exercise was similar for treadmill and cycle ergometry exercises performed in the upright posture. Additionally, we report that as few as four bouts of high‐intensity interval exercise were able to produce rapid plasma volume expansion similar to that seen following eight bouts of high‐intensity exercise.

## AUTHOR CONTRIBUTIONS

W. Bradley Nelson, James M. Walker, and Gary W. Mack conceived and designed the research; W. Bradley Nelson, James M. Walker, Kristopher M. Foote, Nathan A. Bexfield, and Crystelle Hansen performed the experiments; W. Bradley Nelson, James M. Walker, and Gary W. Mack analyzed the data; W. Bradley Nelson and Gary W. Mack wrote the manuscript; W. Bradley Nelson, James M. Walker, Kristopher M. Foote, Nathan A. Bexfield, Crystelle Hansen, and Gary W. Mack edited, revised, and approved the final manuscript.

## ETHICS STATEMENT

Subjects gave written informed consent to the current protocol that was approved by the Brigham Young University Institutional Review Board and conducted in accordance with the Declaration of Helsinki.

## CONFLICT OF INTEREST STATEMENT

The authors have no conflicts of interest to disclose.

## References

[phy215601-bib-0001] Bos, W. J. , Verrij, E. , Vincent, H. H. , Westerhof, B. E. , Parati, G. , & van Montfrans, G. A. (2007). How to assess mean blood pressure properly at the brachial artery level. Journal of Hypertension, 25, 751–755. 17351365 10.1097/HJH.0b013e32803fb621

[phy215601-bib-0002] Brace, R. A. (1989). Effects of outflow pressure on fetal lymph flow. American Journal of Obstetrics and Gynecology, 160, 494–497. 2916638 10.1016/0002-9378(89)90479-1

[phy215601-bib-0003] Brotherhood, J. , Brozovic, B. , & Pugh, L. G. (1975). Haematological status of middle‐ and long‐distance runners. Clinical Science and Molecular Medicine, 48, 139–145. 1116332 10.1042/cs0480139

[phy215601-bib-0004] Convertino, V. A. (1983). Heart rate and sweat rate responses associated with exercise‐induced hypervolemia. Medicine and Science in Sports and Exercise, 15, 77–82. 6843324

[phy215601-bib-0005] Convertino, V. A. (1991). Blood volume: Its adaptation to endurance training. Medicine and Science in Sports and Exercise, 23, 1338–1348. 1798375

[phy215601-bib-0006] Convertino, V. A. , Brock, P. J. , Keil, L. C. , Bernauer, E. M. , & Greenleaf, J. E. (1980). Exercise training‐induced hypervolemia: Role of plasma albumin, renin, and vasopressin. Journal of Applied Physiology: Respiratory, Environmental and Exercise Physiology, 48, 665–669. 6991463 10.1152/jappl.1980.48.4.665

[phy215601-bib-0007] Convertino, V. A. , Keil, L. C. , Bernauer, E. M. , & Greenleaf, J. E. (1981). Plasma volume, osmolality, vasopressin, and renin activity during graded exercise in man. Journal of Applied Physiology: Respiratory, Environmental and Exercise Physiology, 50, 123–128. 7009522 10.1152/jappl.1981.50.1.123

[phy215601-bib-0008] Coyle, E. F. , Hemmert, M. K. , & Coggan, A. R. (1986). Effects of detraining on cardiovascular responses to exercise: Role of blood volume. Journal of Applied Physiology (1985), 60, 95–99. 10.1152/jappl.1986.60.1.95 3944049

[phy215601-bib-0009] Dill, D. B. , & Costill, D. L. (1974). Calculation of percentage changes in volumes of blood, plasma, and red cells in dehydration. Journal of Applied Physiology (1985), 37, 247–248. 10.1152/jappl.1974.37.2.247 4850854

[phy215601-bib-0010] Fortney, S. M. , Wenger, C. B. , Bove, J. R. , & Nadel, E. R. (1983). Effect of blood volume on forearm venous and cardiac stroke volume during exercise. Journal of Applied Physiology: Respiratory, Environmental and Exercise Physiology, 55, 884–890. 6629925 10.1152/jappl.1983.55.3.884

[phy215601-bib-0011] Freund, B. J. , Claybaugh, J. R. , Dice, M. S. , & Hashiro, G. M. (1987). Hormonal and vascular fluid responses to maximal exercise in trained and untrained males. Journal of Applied Physiology (1985), 63, 669–675, 675. 10.1152/jappl.1987.63.2.669 2958440

[phy215601-bib-0012] Gillen, C. M. , Lee, R. , Mack, G. W. , Tomaselli, C. M. , Nishiyasu, T. , & Nadel, E. R. (1991). Plasma volume expansion in humans after a single intense exercise protocol. Journal of Applied Physiology (1985), 71, 1914–1920. 10.1152/jappl.1991.71.5.1914 1761491

[phy215601-bib-0013] Gillen, C. M. , Nishiyasu, T. , Langhans, G. , Weseman, C. , Mack, G. W. , & Nadel, E. R. (1994). Cardiovascular and renal function during exercise‐induced blood volume expansion in men. Journal of Applied Physiology (1985), 76, 2602–2610. 10.1152/jappl.1994.76.6.2602 7928889

[phy215601-bib-0014] Gonzalez‐Alonso, J. , Mora‐Rodriguez, R. , & Coyle, E. F. (2000). Stroke volume during exercise: Interaction of environment and hydration. American Journal of Physiology. Heart and Circulatory Physiology, 278, H321–H330. 10666060 10.1152/ajpheart.2000.278.2.H321

[phy215601-bib-0015] Green, H. J. , Thomson, J. A. , Ball, M. E. , Hughson, R. L. , Houston, M. E. , & Sharratt, M. T. (1984). Alterations in blood volume following short‐term supramaximal exercise. Journal of Applied Physiology: Respiratory, Environmental and Exercise Physiology, 56, 145–149. 6693314 10.1152/jappl.1984.56.1.145

[phy215601-bib-0016] Greenleaf, J. E. , Convertino, V. A. , & Mangseth, G. R. (1979). Plasma volume during stress in man: Osmolality and red cell volume. Journal of Applied Physiology: Respiratory, Environmental and Exercise Physiology, 47, 1031–1038. 389911 10.1152/jappl.1979.47.5.1031

[phy215601-bib-0017] Haskell, A. , Gillen, C. M. , Mack, G. W. , & Nadel, E. R. (1998). Albumin infusion in humans does not model exercise induced hypervolaemia after 24 hours. Acta Physiologica Scandinavica, 164, 277–284. 9853015 10.1046/j.1365-201X.1998.00431.x

[phy215601-bib-0018] Haskell, A. , Nadel, E. R. , Stachenfeld, N. S. , Nagashima, K. , & Mack, G. W. (1997). Transcapillary escape rate of albumin in humans during exercise‐induced hypervolemia. Journal of Applied Physiology (1985), 83, 407–413. 10.1152/jappl.1997.83.2.407 9262434

[phy215601-bib-0019] Hayes, P. M. , Lucas, J. C. , & Shi, X. (2000). Importance of post‐exercise hypotension in plasma volume restoration. Acta Physiologica Scandinavica, 169, 115–124. 10848641 10.1046/j.1365-201x.2000.00728.x

[phy215601-bib-0020] Jacobsson, S. , & Kjellmer, I. (1964). Flow and protein content of lymph in resting and exercising skeletal muscle. Acta Physiologica Scandinavica, 60, 278–285. 14131841 10.1111/j.1748-1716.1964.tb02889.x

[phy215601-bib-0021] Mack, G. W. , Nose, H. , Takamata, A. , Okuno, T. , & Morimoto, T. (1994). Influence of exercise intensity and plasma volume on active cutaneous vasodilation in humans. Medicine and Science in Sports and Exercise, 26, 209–216. 8164538 10.1249/00005768-199402000-00011

[phy215601-bib-0022] Mack, G. W. , Yang, R. , Hargens, A. R. , Nagashima, K. , & Haskell, A. (1998). Influence of hydrostatic pressure gradients on regulation of plasma volume after exercise. Journal of Applied Physiology (1985), 85, 667–675. 10.1152/jappl.1998.85.2.667 9688745

[phy215601-bib-0023] Nagashima, K. , Cline, G. W. , Mack, G. W. , Shulman, G. I. , & Nadel, E. R. (2000). Intense exercise stimulates albumin synthesis in the upright posture. Journal of Applied Physiology (1985), 88, 41–46. 10.1152/jappl.2000.88.1.41 10642360

[phy215601-bib-0024] Nagashima, K. , Mack, G. W. , Haskell, A. , Nishiyasu, T. , & Nadel, E. R. (1999). Mechanism for the posture‐specific plasma volume increase after a single intense exercise protocol. Journal of Applied Physiology (1985), 86, 867–873. 10.1152/jappl.1999.86.3.867 10066698

[phy215601-bib-0025] Nagashima, K. , Wu, J. , Kavouras, S. A. , & Mack, G. W. (2001). Increased renal tubular sodium reabsorption during exercise‐induced hypervolemia in humans. Journal of Applied Physiology (1985), 91, 1229–1236. 10.1152/jappl.2001.91.3.1229 11509520

[phy215601-bib-0026] Nose, H. , Mack, G. W. , Shi, X. R. , & Nadel, E. R. (1988). Shift in body fluid compartments after dehydration in humans. Journal of Applied Physiology (1985), 65, 318–324. 10.1152/jappl.1988.65.1.318 3403475

[phy215601-bib-0027] Olszewski, W. , Engeset, A. , Jaeger, P. M. , Sokolowski, J. , & Theodorsen, L. (1977). Flow and composition of leg lymph in normal men during venous stasis, muscular activity and local hyperthermia. Acta Physiologica Scandinavica, 99, 149–155. 842371 10.1111/j.1748-1716.1977.tb10365.x

[phy215601-bib-0028] Olszewski, W. L. , & Engeset, A. (1980). Intrinsic contractility of prenodal lymph vessels and lymph flow in human leg. The American Journal of Physiology, 239, H775–H783. 7446752 10.1152/ajpheart.1980.239.6.H775

[phy215601-bib-0029] Olszewski, W. L. , Engeset, A. , & Sokolowski, J. (1977). Lymph flow and protein in the normal male leg during lying, getting up, and walking. Lymphology, 10, 178–183. 563502

[phy215601-bib-0030] Ray, C. A. , Cureton, K. J. , & Ouzts, H. G. (1990). Postural specificity of cardiovascular adaptations to exercise training. Journal of Applied Physiology (1985), 69, 2202–2208. 10.1152/jappl.1990.69.6.2202 2077017

[phy215601-bib-0031] Reed, R. K. (1985). Transcapillary extravasation rate of albumin in rat skeletal muscle. Effect of motor activity. Acta Physiologica Scandinavica, 125, 719–725. 4091011 10.1111/j.1748-1716.1985.tb07775.x

[phy215601-bib-0032] Reed, R. K. , Johansen, S. , & Noddeland, H. (1985). Turnover rate of interstitial albumin in rat skin and skeletal muscle. Effects of limb movements and motor activity. Acta Physiologica Scandinavica, 125, 711–718. 4091010 10.1111/j.1748-1716.1985.tb07774.x

[phy215601-bib-0033] Sawka, M. N. , Convertino, V. A. , Eichner, E. R. , Schnieder, S. M. , & Young, A. J. (2000). Blood volume: Importance and adaptations to exercise training, environmental stresses, and trauma/sickness. Medicine and Science in Sports and Exercise, 32, 332–348. 10694114 10.1097/00005768-200002000-00012

[phy215601-bib-0034] Szabo, G. , & Magyar, Z. (1967). Effect of increased systemic venous pressure on lymph pressure and flow. The American Journal of Physiology, 212, 1469–1474. 4952138 10.1152/ajplegacy.1967.212.6.1469

[phy215601-bib-0035] Wu, J. , & Mack, G. W. (2001). Effect of lymphatic outflow pressure on lymphatic albumin transport in humans. Journal of Applied Physiology (1985), 91, 1223–1228. 10.1152/jappl.2001.91.3.1223 11509519

[phy215601-bib-0036] Wyndham, C. H. , Benade, A. J. , Williams, C. G. , Strydom, N. B. , Goldin, A. , & Heyns, A. J. (1968). Changes in central circulation and body fluid spaces during acclimatization to heat. Journal of Applied Physiology, 25, 586–593. 5687365 10.1152/jappl.1968.25.5.586

[phy215601-bib-0037] Wyndham, C. H. , Rogers, G. G. , Senay, L. C. , & Mitchell, D. (1976). Acclimization in a hot, humid environment: Cardiovascular adjustments. Journal of Applied Physiology, 40, 779–785. 931906 10.1152/jappl.1976.40.5.779

[phy215601-bib-0038] Yang, R. C. , Mack, G. W. , Wolfe, R. R. , & Nadel, E. R. (1998). Albumin synthesis after intense intermittent exercise in human subjects. Journal of Applied Physiology (1985), 84, 584–592. 10.1152/jappl.1998.84.2.584 9475869

